# The Oral–Endocrine Interface: Clinical Oral Health Burden, Oral Health‐Related Quality of Life, and Psychosocial Determinants in Women With Polycystic Ovary Syndrome—Evidence for Cross‐Disciplinary Prevention

**DOI:** 10.1155/ijod/9379119

**Published:** 2026-06-30

**Authors:** Jeevan V. Sambasivan, Deepak Kumar Singhal, Nishu Singla, Jishnu Pradeep, Aadil Abdulhameed

**Affiliations:** ^1^ Department of Public Health Dentistry, Manipal College of Dental Sciences, Manipal Academy of Higher Education, Manipal, 576104, India, manipal.edu

**Keywords:** OHRQoL, oral health, perceived stress, polycystic ovary syndrome, SDG 3

## Abstract

**Background:**

This study compared oral health status and oral health‐related quality of life (OHRQoL) in women with polycystic ovary syndrome (PCOS) and age‐matched healthy controls and identified psychosocial and lifestyle determinants of oral health outcomes.

**Materials and Methods:**

A cross‐sectional study enrolled 72 women aged 18–45 years (36 PCOS, 36 controls) from a gynecology outpatient department, with PCOS confirmed using 2003 Rotterdam criteria. Oral health was assessed using decayed, missing, and filled teeth (DMFT) and gingival bleeding score. OHRQoL, psychological stress, and lifestyle quality were measured using Oral Health Impact Profile (OHIP‐14), four‐item Perceived Stress Scale (PSS‐4), and eight‐item Health Practice Index (HPI‐8), respectively. Analysis included Mann–Whitney *U*, Pearson chi‐square, Spearman correlation, logistic, and multiple linear regression.

**Results:**

Both groups were sociodemographically comparable. PCOS women had significantly higher DMFT scores (median 4.00 vs. 1.50; *p*  < 0.001), gingival bleeding scores (median 2.00 vs. 0.00; *p*  < 0.001), and OHIP‐14 total scores (median 8.50 vs. 2.50; *p*  < 0.001) than controls. Six of seven OHIP‐14 domains were significantly elevated; psychological discomfort and psychological disability showed the greatest differences. PSS‐4 and HPI‐8 scores did not differ significantly between groups (*p* = 0.315 and *p* = 0.200, respectively). In an exploratory binary logistic regression, gingival bleeding (OR = 3.010), DMFT (OR = 2.187), and OHIP‐14 total score (OR = 1.630) were independently associated with PCOS status (model accuracy 88.9%; to be interpreted cautiously, given sample size). PCOS disease status was the strongest variable associated with OHRQoL in the full‐sample model (*β* = 0.384, *p*  < 0.001; adjusted *R* ^2^ = 0.589). Within PCOS subgroup, perceived psychological stress was the sole significant independent variable associated with OHRQoL (*β* = 0.428, *p* = 0.009).

**Conclusion:**

Women with PCOS demonstrated significantly poorer oral health and reduced OHRQoL than healthy controls, with perceived psychological stress independently associated with OHRQoL within the PCOS subgroup. These associations are exploratory findings that require confirmation in larger longitudinal studies. Pending such confirmation, the findings support consideration of oral health assessment and psychosocial screening as components of multidisciplinary PCOS management, in alignment with Sustainable Development Goal 3.

**Trial Registration:**

Clinical Trials Registry of India: CTRI/2025/07/090714

## 1. Introduction

Dental caries and periodontal diseases, and their impact on quality of life, have been classified as noncommunicable diseases (NCDs) sharing risk factors with systemic conditions such as diabetes, cardiovascular disease, and endocrine disorders. Although the evidence for the association between oral health and NCDs has existed for decades, the response to this evidence at the policy level has been negligible. Yet, for millions of women living with polycystic ovary syndrome (PCOS) or other NCDs, an integrated policy framework linking oral health prevention to clinical NCD management remains largely unrealized.

PCOS is the most common endocrine and metabolic disorder in women of reproductive age defined by androgen excess and ovarian dysfunction after excluding other disorders [1]. Its clinical features include reproductive symptoms such as infertility, hyperandrogenism, and hirsutism; metabolic disturbances including insulin resistance, impaired glucose tolerance, and increased cardiovascular risk; psychological disturbances and decreased quality of life [2]. Teede et al. [2] highlighted that effective PCOS management requires addressing psychological well‐being, as poor mental health hinders the lifestyle changes essential for treatment. The syndrome affects an estimated 8%–13% of women of reproductive age globally, with Indian prevalence estimates of 4% to 22.5% depending on the diagnostic criteria and population studied [3, 4].

Despite international guidelines advocating prevention‐focused, multidisciplinary PCOS management, such care remains inconsistently implemented in practice. Reviewing the literature, Coffey and Mason [5] found that PCOS patients had significantly higher depression and psychosexual morbidity and stress than the controls, with weight gain and hirsutism identified as their most significant concerns. Despite this breadth of morbidity, healthcare system responses have largely prioritized the metabolic and endocrine aspects of PCOS, with psychosocial and oral health dimensions remaining neglected.

Both PCOS and periodontal health share pathophysiological mechanisms, including systemic low‐grade inflammation, insulin resistance, oxidative stress, and hormone imbalance that establish a biologically plausible and potentially bidirectional relationship between the two conditions [6]. Early systematic evaluations of periodontal status in PCOS demonstrated significantly higher probing depth, gingival index, bleeding on probing, and plaque index scores in women with PCOS compared with age‐ and weight‐matched controls, with elevated gingival crevicular fluid myeloperoxidase, nitric oxide, and malondialdehyde levels indicative of local oxidative stress [7, 8]. Importantly, these findings were documented in nonobese, normoglycemic women [8] and subsequently replicated in an Indian cohort [9].

PCOS and gingival inflammation appear to act synergistically on proinflammatory cytokines, with PCOS women with gingivitis exhibiting significantly higher GCF, salivary, and serum levels of IL‐6 and TNF‐α than both healthy controls and PCOS women with healthy periodontium, suggesting that PCOS exacerbates gingival inflammation through a proinflammatory host state rather than local periodontal factors [10]. Epidemiological evidence corroborates this mechanism, with meta‐analytic data estimating that women with PCOS face a 28% higher risk of periodontal disease, while women with periodontal disease face a 46% higher risk of PCOS [6], a bidirectional relationship attributed to shared pathways [11]. A recent systematic review further identified elevated salivary MMP‐8, IL‐6, CRP, and reactive oxygen species alongside salivary microbiome alterations as markers of heightened periodontal susceptibility in PCOS, concluding that salivary biomarkers represent a promising periodontal risk screening tool in PCOS and calling for collaborative dental–gynecological management [12].

The management of periodontal disease in systemically affected populations has increasingly incorporated regenerative approaches; biocompatible biomaterials, including bovine pericardium membranes, have demonstrated capacity to support periodontal ligament fibroblast attachment in guided tissue regeneration, representing a complementary avenue for oral disease management in populations such as women with PCOS whose elevated periodontal susceptibility has been documented [13]. Despite growing evidence linking PCOS to periodontal disease and gingival inflammation, no published study has assessed oral health‐related quality of life (OHRQoL) in this population using a validated oral health‐specific instrument. OHRQoL captures the functional, psychological, and social impact of oral disorders on individual well‐being. The Oral Health Impact Profile (OHIP‐14), comprising 14 items across seven domains, is the most widely used instrument for this purpose. Previous studies of quality of life in PCOS have used generic questionnaires such as the SF‐36 or the PCOSQ specific for the condition, which do not include the oral health dimension. Coffey and Mason [5] further underscored the inadequacy of PCOS‐specific instruments in fully capturing the psychosocial burden of the condition, stressing the importance of validated multidimensional assessment.

A further knowledge gap concerns the role of psychosocial and lifestyle factors in determining oral health outcomes in PCOS. Perceived psychological stress is significantly associated with unhealthy behaviors and neuroendocrine disturbances in PCOS [2] and may plausibly influence oral health through dental attendance, diet, and immune function. Singla et al. [14] demonstrated that poor lifestyle quality was an independent risk factor for periodontal disease in an Indian adult population. No study has investigated the specific role of perceived stress and lifestyle quality in determining OHRQoL in PCOS patients or compared these determinants with healthy controls.

This gap is clinically and policy‐relevant. PCOS affects a substantial proportion of women during their reproductive years, and its management guidelines increasingly advocate for multidisciplinary approaches that address metabolic, reproductive, and psychological features simultaneously [2]. Evidence linking PCOS to impaired oral health and OHRQoL and identifying psychosocial and lifestyle determinants of this association would strengthen the case for integrating oral health assessment into routine PCOS management. Conversely, dental professionals identifying young women with unexplained gingival inflammation or oral health‐related psychological distress may be well‐placed to facilitate gynecological referral. The policy case for cross‐disciplinary integration is strengthened when the oral health burden of PCOS is quantified with validated instruments and linked to identifiable, modifiable psychosocial determinants. The present study was therefore designed to address three objectives in a comparative cross‐sectional design involving women with PCOS and age‐matched healthy controls. First, to compare the oral health status of women with PCOS and healthy women, as measured by decayed, missing, and filled teeth (DMFT) scores and gingival bleeding. Second, compare OHRQoL between women with PCOS and healthy controls using the OHIP‐14. Third, to evaluate the lifestyle and psychosocial factors associated with PCOS and oral health outcomes and to identify variables independently associated with OHRQoL in the full sample and within the PCOS subgroup.

## 2. Materials and Methods

A comparative cross‐sectional study was conducted in the Department of Public Health Dentistry, Manipal College of Dental Sciences (MCODS), Manipal Academy of Higher Education (MAHE), Manipal, in collaboration with the Department of Obstetrics and Gynecology, Dr. TMA Pai Hospital, Udupi. Institutional ethics committee clearance was obtained from the MCODS Institutional Review Committee and from the Kasturba Medical College and Kasturba Hospital Institutional Ethics Committee (IEC‐2), Manipal, prior to the commencement of data collection. Permission to conduct dental examinations within the Department of Obstetrics and Gynecology was obtained from the Medical Superintendent, Dr. TMA Pai Hospital, Udupi.

The study population comprised women aged 18–45 years attending the gynecology outpatient department. Two groups were recruited: Group 1 (PCOS group), consisting of women with a confirmed diagnosis of PCOS established by a qualified gynecologist on the basis of the 2003 Rotterdam criteria, requiring the presence of at least two of the following: oligoovulation and/or anovulation, clinical and/or biochemical signs of hyperandrogenism, and polycystic ovarian morphology on ultrasound, in the absence of other specific diagnoses; and Group 2 (control group), consisting of healthy women, aged 18–45, with no history of PCOS and regular menstrual cycles. Women were eligible for the PCOS group if they had confirmed PCOS, irregular menstrual cycles and were willing to provide written informed consent. Participants were excluded if they were pregnant or breastfeeding, had a history of conditions that may mimic PCOS (including congenital adrenal hyperplasia, Cushing’s syndrome, or androgen‐secreting tumors), had a history of ovarian cancer or surgery affecting ovarian function, had a history of osteoporosis, had a body mass index (BMI) exceeding 25 kg/m^2^, in order to minimize the confounding effect of obesity on periodontal and metabolic outcomes, were differently abled or receiving psychotropic medication, or declined to provide informed consent.

The sample size was determined from a preliminary pilot study of 20 participants (10 in each group). Based on the mean OHIP‐14 total scores obtained in the pilot—5.0 in the PCOS group and 2.3 in the control group, and a Cohen’s *d* effect size of 0.66, a minimum sample size of 36 participants per group was calculated at 80% statistical power and a significance level of 0.05. The final study enrolled 36 women with PCOS and 36 age‐matched healthy controls, yielding a total sample of 72 participants.

Data collection consisted of clinical assessment of oral health status and patient‐reported outcome measures. Clinical examination was performed using the World Health Organization Oral Health Assessment Form, Version 2013, [15]. The DMFT index was recorded to assess the cumulative burden of dental caries. Gingival bleeding was assessed using the sextant‐based bleeding score, with the number of sextants exhibiting gingival bleeding on the examination recorded for each participant. Periodontal status was additionally assessed using the Community Periodontal Index (CPI) [15]. Given that CPI scores in the study population were confined to the range indicating healthy periodontium or gingivitis, the sextant‐based gingival bleeding score was identified as the clinically appropriate primary periodontal outcome measure and was used in all subsequent analyses. All clinical examinations were conducted by a single trained examiner (JVS) who underwent calibration prior to data collection. Intraexaminer reliability was assessed using Cohen’s kappa for gingival bleeding scores and intraclass correlation coefficient (ICC) for DMFT scores on a subset of 10 participants examined on two occasions. Kappa and ICC values exceeded 0.80, indicating strong intraexaminer agreement. Patient‐reported outcome measures were collected via a self‐administered questionnaire in Kannada or English according to participant preference. Kannada translations of all instruments were prepared and validated using a back‐translation method, in which blind retranslation into English was verified by bilingual subject‐matter experts. OHRQoL was assessed using the OHIP‐14, a widely validated instrument comprising 14 items across seven domains: functional limitation, physical pain, psychological discomfort, physical disability, psychological disability, social disability, and handicap [16, 17]. Each item was scored on a five‐point Likert scale, with higher total scores indicating greater oral health impact. Perceived psychological stress was measured using the four‐item Perceived Stress Scale (PSS‐4), a validated brief instrument designed to assess the degree to which respondents appraise their life circumstances as unpredictable, uncontrollable, and overloading [18]. Lifestyle quality was assessed using the eight‐item Health Practice Index (HPI‐8), developed by Morimoto [19], which captures information on eight health‐related behaviors: smoking, alcohol consumption, breakfast habits, hours of sleep per night, hours of work per day, physical exercise, nutritional balance, and mental stress management. A standardized questionnaire was used to gather sociodemographic data, such as age, educational attainment, occupation, and monthly income. All participants’ anthropometric measurements, such as height, weight, and BMI, were noted.

Statistical analysis was performed using IBM SPSS Statistics, version 23.0. Continuous variables were reported as the median and interquartile range (IQR) given the nonnormal distribution of most outcome variables, as assessed by inspection of distributional properties. The Mann–Whitney *U* test was used to compare continuous variables between the PCOS and control groups. Categorical variables were compared using the Pearson chi‐square test. Spearman rank correlation analysis was conducted to examine the relationships between continuous independent variables and OHIP‐14 total score and to assess intervariable collinearity. Three regression models were constructed following univariate screening; variables attaining *p*  < 0.20 in group comparisons were eligible for inclusion. A binary logistic regression model was used to identify variables independently associated with PCOS status, with PCOS versus control as the dependent variable. Two multiple linear regression models were constructed with the OHIP‐14 total score as the dependent variable: one in the full sample and one restricted to the PCOS subgroup. Model size was constrained in accordance with the recommended events‐per‐variable ratio. Multicollinearity was assessed using variance inflation factors (VIF), with VIF values below 10 considered acceptable. Model fit for logistic regression was assessed using the Hosmer–Lemeshow test. The level of statistical significance was set at *p*  < 0.05 (two‐tailed) for all analyses. Prior to regression analysis, continuous outcome variables were examined for normality using the Shapiro–Wilk test. The OHIP‐14 total score demonstrated a right‐skewed distribution, consistent with the use of nonparametric tests for between‐group comparisons; however, given that multiple linear regression is moderately robust to nonnormality of the dependent variable with sample sizes above 30, and given that the residuals of the fitted regression models were approximately normally distributed upon inspection, parametric regression was considered appropriate.

Variable selection for regression models was guided by a two‐step process: first, univariate screening using Mann–Whitney *U* or chi‐square tests, with variables attaining *p*  < 0.20 considered eligible for inclusion; second, Spearman correlation analysis to assess intervariable relationships and identify potential multicollinearity. Disease status was included a priori in Model 2 as the primary variable of interest.

## 3. Results

### 3.1. Participant Characteristics

A total of 72 female participants were enrolled, comprising 36 women with a confirmed diagnosis of PCOS and 36 age‐matched healthy controls. The two groups were comparable with respect to age (both medians: 26 years; *p* = 0.598), BMI (control: 22.13 kg/m^2^, PCOS: 22.86 kg/m^2^; *p* = 0.111), marital status (*p* = 0.624), occupation (*p* = 0.752), and annual income (*p* = 0.371), confirming successful matching and sociodemographic equivalence. Continuous and categorical group comparisons are presented in Tables 1 and 2, respectively.

**Table 1:** Comparison of continuous variables between PCOS and control groups.

Variable	Control (*n* = 36) median (IQR)	PCOS (*n* = 36) median (IQR)	*p*‐Value
Demographic variables			
Age (years)	26.00 (5.00)	26.00 (4.00)	0.598
BMI (kg/m^2^)	22.13 (3.25)	22.86 (2.63)	0.111

Oral health clinical parameters			
DMFT score	1.50 (3.75)	4.00 (3.00)	<0.001 ^∗∗^
Gingival bleeding (sextants)	0.00 (0.75)	2.00 (2.00)	<0.001 ^∗∗^

OHRQoL—OHIP‐14 total and domain scores			
OHIP‐14 total score (0–56)	2.50 (5.00)	8.50 (4.75)	<0.001 ^∗∗^
Functional limitation	0.00 (0.00)	0.00 (1.00)	0.010 ^∗^
Physical pain	0.00 (2.00)	2.00 (2.00)	0.064
Psychological discomfort	0.00 (2.00)	3.50 (3.00)	<0.001 ^∗∗^
Physical disability	0.00 (0.00)	0.00 (1.00)	0.032 ^∗^
Psychological disability	0.00 (0.00)	2.00 (2.75)	<0.001 ^∗∗^
Social disability	0.00 (0.00)	0.00 (1.75)	0.002 ^∗∗^
Handicap	0.00 (0.00)	0.00 (1.00)	0.017 ^∗^

Psychosocial and lifestyle variables			
PSS‐4 total score (0–16)	7.00 (2.75)	8.00 (2.75)	0.315
HPI‐8 total score (0–8)	5.00 (2.50)	5.00 (2.75)	0.200

*Note:* Values expressed as median (IQR). Mann–Whitney *U* test.

Abbreviations: BMI, body mass index; DMFT, decayed‐missing‐filled teeth; HPI‐8, Health‐Promoting Index‐8; IQR, interquartile range; OHIP‐14, Oral Health Impact Profile‐14; OHRQoL, oral health‐related quality of life; PSS‐4, Perceived Stress Scale‐4.

^∗^ *p*  < 0.05.

^∗∗^ *p*  < 0.01.

**Table 2:** Comparison of categorical variables between PCOS and control groups.

Variable	Control *n* (%)	PCOS *n* (%)	*p*‐Value
Marital status			
Not married	22 (61.1%)	24 (66.7%)	0.624
Married	14 (38.9%)	12 (33.3%)	—

Occupation			
Student	22 (61.1%)	21 (58.3%)	0.752
Professional	10 (27.8%)	12 (33.3%)	—
Not working	3 (8.3%)	3 (8.3%)	—
Semiskilled	1 (2.8%)	0 (0.0%)	—

Annual income			
NIL (student/nonearning)	23 (63.9%)	24 (66.7%)	0.371
Low–middle income	9 (25.0%)	5 (13.9%)	—
High income	4 (11.1%)	7 (19.4%)	—

*Note:* Values expressed as *n* (%). Pearson chi‐square test. *p*‐Values correspond to overall group comparison.

### 3.2. Oral Health Status and OHRQoL

#### 3.2.1. Clinical Oral Health Parameters

DMFT scores were significantly higher in PCOS women (median: 4.00, IQR: 3.00) than controls (median: 1.50, IQR: 3.75; *p*  < 0.001). Similarly, gingival bleeding scores were significantly higher in the PCOS group (median: 2.00, IQR: 2.00) than in controls (median: 0.00, IQR: 0.75; *p*  < 0.001) (Table 1). CPI assessment confirmed that no participant in either group presented with periodontal pocketing; CPI scores were limited to 0 (healthy periodontium), 1 (bleeding on probing), and 2 (calculus detected) across all 72 participants, establishing that the observed gingival inflammatory burden in both groups represented gingivitis rather than periodontitis.

#### 3.2.2. OHRQoL

OHIP‐14 total scores were significantly higher in the PCOS group (median: 8.50, IQR: 4.75) than in controls (median: 2.50, IQR: 5.00; *p*  < 0.001). Of the seven OHIP domains, six differed significantly between groups: psychological discomfort (*p*  < 0.001), psychological disability (*p*  < 0.001), social disability (*p* = 0.002), functional limitation (*p* = 0.010), handicap (*p* = 0.017), and physical disability (*p* = 0.032). Physical pain did not reach a statistically significant difference (*p* = 0.064) (Table 1).

#### 3.2.3. Psychosocial and Lifestyle Variables

No statistically significant between‐group differences were observed for PSS‐4 total scores (control median: 7.00 vs. PCOS median: 8.00; *p* = 0.315) or HPI‐8 total scores (both groups median: 5.00; *p* = 0.200) (Table 1).

### 3.3. Spearman Correlation Analysis

Spearman correlation analysis identified gingival bleeding as the strongest correlate of OHIP‐14 total score (*r* = 0.617, *p*  < 0.001), followed by DMFT (*r* = 0.411, *p*  < 0.001). PSS‐4 (*r* = 0.254, *p* = 0.032) and HPI‐8 (*r* = −0.254, *p* = 0.031) each demonstrated weak but statistically significant correlations with OHIP‐14 total score; the negative coefficient for HPI‐8 indicated that higher lifestyle scores were associated with lower OHIP‐14 scores. BMI demonstrated a near‐zero, non‐significant correlation with OHIP‐14 total score (*r* = 0.038, *p* = 0.749). No intervariable correlation exceeded *r* = 0.323 (DMFT vs. gingival bleeding, *p* = 0.006), below the accepted multicollinearity threshold of *r* = 0.80. Detailed correlation coefficients are presented in Table 3.

**Table 3:** Spearman correlation analysis of continuous variables with OHIP‐14 total score.

Variable pair	Spearman *r*	*p*‐Value	Interpretation
Variables vs. OHIP‐14 total score			
DMFT vs. OHIP‐14 total	0.411 ^∗∗^	<0.001	Moderate positive
Gingival bleeding vs. OHIP‐14 total	0.617 ^∗∗^	<0.001	Strong positive
PSS‐4 vs. OHIP‐14 total	0.254 ^∗^	0.032	Weak positive
HPI‐8 vs. OHIP‐14 total	−0.254 ^∗^	0.031	Weak inverse
BMI vs. OHIP‐14 total	0.038	0.749	Negligible

Intervariable correlations (multicollinearity check)			
DMFT vs. gingival bleeding	0.323 ^∗∗^	0.006	Low risk
All other variable pairs	<0.32	>0.14	No risk

*Note: r*, Spearman rho correlation coefficient.

Abbreviations: DMFT, decayed‐missing‐filled teeth; HPI‐8, Health‐Promoting Index‐8; OHIP‐14, Oral Health Impact Profile‐14; PSS‐4, Perceived Stress Scale‐4.

^∗^ *p*  < 0.05.

^∗∗^ *p*  < 0.01 (two‐tailed).

### 3.4. Regression Analyses

#### 3.4.1. Model 1: Binary Logistic Regression—Variables Independently Associated With PCOS Status

Binary logistic regression with PCOS status as the dependent variable and DMFT, gingival bleeding, OHIP‐14 total score, BMI, and HPI‐8 as covariates yielded a statistically significant model (*χ* ^2^ = 62.853, df = 5, *p*  < 0.001; Nagelkerke *R* ^2^ = 0.776). The Hosmer–Lemeshow test confirmed adequate fit (*χ* ^2^ = 1.572, *p* = 0.991). Overall classification accuracy in this exploratory model was 88.9% (sensitivity 86.1%, specificity 91.7%); three variables were independently associated with PCOS status in the model: OHIP‐14 total score (OR = 1.630, 95% CI: 1.181–2.250, *p* = 0.003), DMFT (OR = 2.187, 95% CI: 1.213–3.945, *p* = 0.009), and gingival bleeding (OR = 3.010, 95% CI: 1.134–7.989, *p* = 0.027). BMI (*p* = 0.067) and HPI‐8 (*p* = 0.844) were not independently associated with PCOS status (Table 4); this estimate should be interpreted cautiously given the modest sample size and the risk of overfitting in a small sample.

**Table 4:** Binary logistic regression—variables independently associated with PCOS status (*n* = 72).

Variable	OR (95% CI)	*p*‐Value
**DMFT score**	2.187 (1.213–3.945)	0.009 ^∗∗^
**Gingival bleeding**	3.010 (1.134–7.989)	0.027 ^∗^
**OHI** **P-14 total score**	1.630 (1.181–2.250)	0.003 ^∗∗^
BMI	1.720 (0.964–3.069)	0.067
HPI‐8 total score	0.937 (0.488–1.797)	0.844

*Note:* Dependent variable: PCOS status (0 = control, 1 = PCOS). Model: *χ* ^2^ = 62.853, *p*  < 0.001; Nagelkerke *R* ^2^ = 0.776; overall accuracy 88.9%. This is an exploratory model; the events‐per‐variable ratio of 7.2 falls below the recommended threshold of 10. Bold rows denote statistical significance.

^∗^ *p*  < 0.05.

^∗∗^ *p*  < 0.01.

#### 3.4.2. Model 2: Multiple Linear Regression—Variables Associated With OHRQoL in the Full Sample

Multiple linear regression with OHIP‐14 total score as the dependent variable and disease status, DMFT, gingival bleeding, PSS‐4, and HPI‐8 as independent variables produced a statistically significant model (*F* [5,66] = 21.367, *p*  < 0.001; adjusted *R* ^2^ = 0.589). BMI was excluded on the basis of its negligible correlation with OHIP‐14 total score (*r* = 0.038, *p* = 0.749). No multicollinearity was detected (all VIF < 2.0). Disease status (PCOS vs. control) was the strongest independent variable associated with OHRQoL (*B* = 3.272, *β* = 0.384, 95% CI: 1.497–5.047, *p*  < 0.001), followed by gingival bleeding (*B* = 1.088, *β* = 0.297, 95% CI: 0.418–1.758, *p* = 0.002), DMFT (*B* = 0.394, *β* = 0.199, 95% CI: 0.030–0.759, *p* = 0.035), and HPI‐8 (*B* = −0.524, *β* = −0.186, 95% CI: −0.984 to −0.065, *p* = 0.026). PSS‐4 did not achieve statistical significance at the prespecified threshold (*B* = 0.334, *β* = 0.156, 95% CI: −0.002 to 0.670, *p* = 0.051) (Table 5), though the magnitude of the association and its confidence interval are of potential clinical interest and warrant examination in larger samples. The adjusted *R* ^2^ of 0.589, while suggesting substantial explained variance, should be interpreted in the context of the modest sample size and the exploratory nature of the analysis.

**Table 5:** Multiple linear regression—variables associated with OHRQoL in the full sample (*n* = 72).

Variable	*B* (95% CI)	*β*	*p*‐Value^a^
**Disease status (PCOS)**	3.272 (1.497–5.047)	0.384	<0.001 ^∗∗^
**Gingival bleeding**	1.088 (0.418–1.758)	0.297	0.002 ^∗∗^
**DMFT score**	0.394 (0.030–0.759)	0.199	0.035 ^∗^
**HPI-8 total score**	−0.524 (−0.984 to −0.065)	−0.186	0.026 ^∗^
PSS‐4 total score	0.334 (−0.002 to 0.670)	0.156	0.051

*Note:* Dependent variable: OHIP‐14 total score. Model: *F* (5,66) = 21.367, *p*  < 0.001; adjusted *R* ^2^ = 0.589. No multicollinearity detected; all VIF values <2.0. Disease status was the strongest variable associated with OHRQoL. Adjusted *R* ^2^ should be interpreted cautiously given the exploratory nature of the analysis. Bold rows denote statistical significance. *B*, unstandardized coefficient; *β*, standardized coefficient.

Abbreviation: CI, confidence interval.

^a^Approached but did not reach the prespecified significance threshold of *p* < 0.05; confidence interval narrowly crosses zero.

^∗^ *p*  < 0.05.

^∗∗^ *p*  < 0.01.

#### 3.4.3. Model 3: Multiple Linear Regression—Variables Associated With OHRQoL in the PCOS Subgroup

A subgroup multiple linear regression within the PCOS group (*n* = 36), with PSS‐4, HPI‐8, and BMI as independent variables in the model for OHIP‐14 total score, was statistically significant (*F* [3,32] = 3.587, *p* = 0.024; adjusted *R* ^2^ = 0.182). The modest adjusted *R* ^2^ of 0.182 is consistent with the restricted variance within a single diagnostic group and the limited number of variables entered and should be interpreted accordingly. No multicollinearity was identified (all VIF < 1.3). PSS‐4 was the sole significant independent variable associated with OHIP‐14 total score (*B* = 0.801, β = 0.428, 95% CI: 0.211–1.391, *p* = 0.009). HPI‐8 showed a negative but non‐significant association (*B* = −0.572, *β* = −0.245, *p* = 0.152), and BMI did not contribute to the model (*B* = 0.054, *β* = 0.029, *p* = 0.863) (Table 6).

**Table 6:** Multiple linear regression—variables associated with OHRQoL in the PCOS subgroup (*n* = 36).

Variable	*B* (95% CI)	*β*	*p*‐Value
**PSS-4 total score**	0.801 (0.211–1.391)	0.428	0.009 ^∗∗^
HPI‐8 total score	−0.572 (−1.366 to 0.221)	−0.245	0.152
BMI	0.054 (−0.583 to 0.691)	0.029	0.863

*Note:* Dependent variable: OHIP‐14 total score (PCOS group only). Model: *F* (3,32) = 3.587, *p* = 0.024; adjusted *R* ^2^ = 0.182. PSS‐4 was the sole significant variable associated with OHRQoL within the PCOS subgroup. No multicollinearity detected; all VIF values <1.3. Bold row denotes statistical significance. *B*, unstandardized coefficient; *β*, standardized coefficient.

Abbreviations: CI, confidence interval; HPI‐8, Health‐Promoting Index‐8; PSS‐4, Perceived Stress Scale‐4.

^∗∗^ *p*  < 0.01.

## 4. Discussion

The aim of the present study was to investigate the oral health status, OHRQoL, and associated psychosocial and lifestyle factors in women with PCOS compared with age‐matched healthy controls. The main findings were that PCOS was independently associated with significantly worse clinical oral health (higher DMFT scores and gingival bleeding) and considerably lower OHRQoL across several OHIP‐14 domains. Exploratory regression modeling identified PCOS disease status, gingival bleeding, DMFT, and lifestyle quality as variables independently associated with OHRQoL in the full sample. Perceived psychological stress was the only significant variable associated with OHRQoL in the PCOS subgroup. All regression findings are exploratory and hypothesis‐generating and need to be replicated in larger prospective cohorts.

### 4.1. Oral Health Status: Dental Caries and Gingival Bleeding

Women with PCOS had significantly higher DMFT scores than those of controls, indicating a higher cumulative burden of dental caries. This finding is consistent with the more complete understanding of PCOS as a multisystem disorder with metabolic, inflammatory, and endocrine sequelae that extend to the oral cavity. Escobar‐Morreale [1] described PCOS as a complex multigenic disorder with strong environmental influences. The hormonal disturbances characteristic of the syndrome, especially hyperandrogenism and insulin resistance, create a systemic inflammatory milieu that may alter the host susceptibility to oral disease [1]. High levels of androgens in PCOS have been associated with changes in salivary flow and composition, both of which influence the risk of caries, and insulin resistance has been proposed as a potential factor that impairs glucose regulation in gingival tissue, providing a local environment conducive to cariogenic bacterial activity [1, 2]. The bidirectional association between systemic metabolic dysregulation and oral health, well described in the context of diabetes and caries, can happen through similar mechanisms in PCOS, especially with the high prevalence of insulin resistance, described in 50%–80% of affected women, documented [2].

Corroborating evidence for a caries‐specific pathway in PCOS was reported by Hilaloglu et al. [20], as synthesized in the recent systematic review by Angiolani et al. [12], which demonstrated that elevated *S. mutans* levels and DMFT scores in PCOS were linked to salivary microbiome alterations driven by insulin resistance and elevated estrogen levels, providing a biologically plausible mechanistic basis for the elevated DMFT observed in the present study.

Gingival bleeding scores were also significantly elevated in PCOS women, independent of BMI and other potential confounders. This finding replicates and extends the observations of Dursun et al. [7], who reported the first systematic evaluation of periodontal status in PCOS and documented significantly higher probing depth, gingival index, bleeding on probing, and plaque index scores in 25 nonobese, normoglycemic women with PCOS compared with age‐ and weight‐matched controls. Researchers from India subsequently confirmed these findings in an Indian cohort, reporting significantly elevated modified gingival index, plaque index, and bleeding on probing in 30 PCOS women relative to controls, with the gingival inflammation observed independently of plaque‐retentive factors and conventional metabolic risk variables [9]. The present study extends these data to a larger, sociodemographically well‐matched sample. It should be noted, however, that plaque index and direct oral hygiene practice data were not analyzed in this study; accordingly, the contribution of differential oral hygiene behavior to the observed gingival bleeding differences cannot be fully excluded, and the association between PCOS and elevated gingival bleeding should be interpreted with caution.

A plausible biological basis for the elevated gingival bleeding in PCOS is the state of chronic low‐grade systemic inflammation intrinsic to the syndrome. Özçaka et al. [10] demonstrated that PCOS and gingivitis act synergistically to amplify gingival concentrations of IL‐6 and TNF‐α, with PCOS women with concurrent gingivitis exhibiting higher serum, salivary, and gingival crevicular fluid concentrations of proinflammatory cytokines than both healthy controls and PCOS women with healthy periodontium. Similarly, Dou et al. [6] reviewed accumulating epidemiological evidence and estimated that women with PCOS face a 28% higher risk of periodontal disease, while women with periodontal disease face a 46% higher risk of PCOS, consistent with a bidirectional inflammatory relationship between the two conditions. These results were confirmed by the systematic review of Márquez‐Arrico [11], which concluded that patients with PCOS are more prone to develop periodontal disease due to a proinflammatory host state. The present study findings are consistent with this more general mechanistic framework. It is important to note that CPI assessment confirmed the absence of periodontal pocketing among all 72 participants in both groups, with CPI scores limited to 0, 1, and 2.

This establishes that the elevated gingival bleeding observed in PCOS women in the present study reflects a heightened gingival inflammatory response consistent with gingivitis rather than established periodontitis. This finding is consistent with the young age of the study population, validates the use of the sextant‐based gingival bleeding score as the appropriate periodontal outcome measure and provides a clear terminological boundary for interpreting the present findings: the study’s results pertain specifically to gingival inflammation and should not be generalized to periodontitis.

### 4.2. OHRQoL

PCOS women reported significantly worse OHRQoL on the OHIP total score and on six of the seven OHIP domains. The domains of psychological discomfort and psychological disability showed the greatest group differences, with PCOS women reporting markedly higher scores than those of controls. The pattern suggests that the burden of oral health in PCOS is not limited to clinically measurable tissue damage but augments into the psychosocial sphere, encompassing worry, self‐consciousness, and interpersonal difficulty caused by oral conditions. Coffey and Mason [5] found elevated levels of psychological morbidity, psychosexual dysfunction, and lower self‐esteem in women with PCOS, attributing these to the syndrome’s visible manifestations, including acne, hirsutism, and weight changes. The present data indicate that oral health problems may be an additional underappreciated contributor to this psychological burden. Researchers also noted that the negative impact of PCOS on feminine identity and body image influences mood and psychological well‐being and that the psychosocial aspects are an important part of PCOS management [2]. The predominance of psychological domain impairment in the OHIP‐14 profile of PCOS women in the present study is consistent with this literature and adds a specifically oral health dimension to the known psychosocial morbidity of the condition.

The findings are noteworthy in that they demonstrate a functional OHRQoL impact in a relatively young, nonobese, and sociodemographically matched sample. Prior OHRQoL research in PCOS has focused predominantly on general health‐related quality of life using instruments such as the SF‐36 or the condition‐specific PCOSQ, with relatively little attention to oral health‐specific quality of life. The present study contributes to an emerging evidence base by demonstrating that oral health is a domain through which a measurable quality‐of‐life penalty is observed in association with PCOS, independent of other known determinants such as BMI and socioeconomic status.

### 4.3. Psychosocial and Lifestyle Variables

In the between‐group comparison, neither PSS‐4 scores nor HPI‐8 scores differed significantly between PCOS women and controls. This finding may appear counterintuitive given the documented psychological burden of PCOS but may reflect the successful matching of groups on key sociodemographic variables, including occupation, education, and income, which are established determinants of perceived stress and health behavior. The absence of a significant group difference in PSS‐4 is not inconsistent with the literature; several studies have noted that psychological outcomes in PCOS are heterogeneous and context‐dependent, varying substantially with the ethnic background, healthcare setting, and individual coping resources [2, 5]. It is plausible that the level of awareness and adjustment to the diagnosis, combined with similar socioeconomic status between groups, attenuated any between‐group stress differential in the present cross‐sectional design.

By contrast, within the PCOS subgroup, perceived psychological stress was the sole statistically significant independent variable associated with OHRQoL (*β* = 0.428, *p* = 0.009), accounting for a meaningful proportion of the within‐group variance even after controlling for BMI and lifestyle quality. This finding is noteworthy as it is consistent with the possibility that stress‐related pathways may contribute to the association between PCOS and oral health impact, potentially through effects on oral health behaviors such as dental attendance, dietary habits, and self‐care, as well as through neuroendocrine and immunological mechanisms; however, mediation cannot be established from cross‐sectional data. Singla et al. [14] in a cross‐sectional study on the lifestyle and periodontal health of adults in the Udupi district demonstrated that overall poor lifestyle (adjusted OR = 2.77, 95% CI: 1.01–7.61) was independently associated with periodontal disease and that physical activity and sleep quality were significant modifying factors. While the present study’s HPI‐8 instrument captured a similar range of health behaviors, its association with OHRQoL was attenuated in the multivariate model, suggesting that it is the perceived psychological appraisal of stress, rather than behavioral lifestyle practices per se, that most directly shapes oral health impact within this population of PCOS‐affected women.

### 4.4. Regression Analyses: Variables Associated With OHRQoL and PCOS Status

An exploratory binary logistic regression showed that gingival bleeding (OR = 3.010), DMFT (OR = 2.187), and OHIP‐14 total score (OR = 1.630) were significantly and independently associated with the PCOS status, with an overall accuracy of 88.9%. This performance estimate should be interpreted with caution given the small sample size and risk of overfitting.

The association of oral clinical parameters with PCOS status is consistent with the growing body of evidence suggesting a bidirectional relationship between oral and reproductive health. One review of this evidence concluded that PCOS and periodontal disease share several pathophysiological mechanisms such as systemic low‐grade inflammation, oxidative stress, insulin resistance, and hormonal imbalance and that there is strong support for a bidirectional link between the two conditions [6]. The association of the OHIP‐14 total score with PCOS status in the logistic model implies that the women’s subjective oral health impact may mirror the underlying PCOS‐related pathophysiology. Whether this association could be considered a screening signal in dental settings warrants prospective investigation.

The strongest independent variable associated with OHRQoL was PCOS disease status, followed by gingival bleeding, DMFT, and HPI‐8 (negatively) in the multiple linear regression of OHRQoL in the full sample. The negative coefficient for HPI‐8 indicated that improved lifestyle practices were independently associated with improved OHRQoL, which is in agreement with the study of Singla et al. [14] that reported a direct association between healthy lifestyle composite scores and superior periodontal outcomes. The model explained a total variance of 0.589 (adjusted *R* ^2^), indicating that PCOS disease status, gingival inflammatory burden, dental caries burden, and lifestyle quality collectively explain a large proportion of the variance of OHRQoL in this exploratory analysis. Interventions aimed at these modifiable factors may have oral health benefits, but this needs to be confirmed in prospective studies.

### 4.5. Group Comparability and the Role of BMI

Both groups were comparable on age, BMI, and all sociodemographic variables, supporting the internal validity of group comparisons. The absence of a significant BMI difference and the near‐zero correlation between BMI and OHIP‐14 total score (*r* = 0.038, *p* = 0.749), suggests that the observed oral health differences between groups reflect PCOS‐specific biological mechanisms rather than adiposity, although residual confounding by unmeasured behavioral variables, including oral hygiene practices and dental attendance, cannot be excluded.

### 4.6. Strengths of the Study

The present study has several methodological strengths. Age matching between groups controlled for the influence of age on both periodontal health and OHRQoL. Confirmation of the PCOS diagnosis by a qualified gynecologist using the 2003 Rotterdam criteria ensured diagnostic accuracy.

The use of standard and validated tools to measure OHRQoL (OHIP‐14) [16, 17], perceived stress (PSS‐4) [18], and lifestyle (HPI‐8) [19] enables comparison with previous studies. All clinical examinations were conducted by a single trained and calibrated examiner, with intraexaminer reliability formally assessed on a subset of 10 participants examined on two occasions; Cohen’s kappa for gingival bleeding scores and ICC for DMFT both exceeded 0.80, confirming strong measurement consistency and minimizing the risk of systematic examiner‐introduced variability in the clinical outcome data.

### 4.7. Limitations of the Study

A few limitations of the study should be considered. First, the cross‐sectional nature of the study precludes any inferences regarding causality. The cause–effect relationship between PCOS, worsening of oral health status, and/or deterioration in OHRQoL cannot be established based on the present study. Second, the sample size (*n* = 72) is relatively small for conducting subgroup analysis and for multiple regression analyses. Future large‐scale prospective studies should be conducted to confirm the findings of the present study. Third, the present study was conducted in a single tertiary health center. This may lead to selection bias because the population visiting a specialized gynecology clinic may not be representative of the general population of women with PCOS in terms of the severity of symptoms and/or health‐related behaviors. Fourth, patient‐reported outcome measures including the OHIP‐14, PSS‐4, and HPI‐8 are subject to social desirability bias, recall bias, and response set effects inherent to the self‐report methodology. While all three instruments are validated and widely used in the literature, their use in a clinical outpatient setting may have introduced systematic response bias, particularly if participants perceived a connection between their responses and their clinical care. Last, hormonal analyses such as testosterone, SHBG, insulin, and markers of oxidative stress were not evaluated in the present study. Therefore, the direct effect of hormonal imbalance on oral health‐related outcomes could not be established. Finally, the study did not assess dental attendance patterns or oral hygiene behaviors, which are important confounders of both caries and gingival outcomes.

CPI assessment confirmed the absence of periodontal pocketing across all participants, limiting the periodontal findings to gingivitis; future studies should incorporate full periodontal staging, including pocket depth, clinical attachment level, and radiographic bone loss.

Another important limitation is the absence of analyzed data on several important behavioral confounders. Dietary habits, especially the frequency of sugar consumption and the quality of diet, which are known determinants of both dental caries and gingival health, were not assessed. Similarly, dental attendance behavior, a strong determinant of both DMFT and gingival health, was not captured. The lack of these behavioral covariates means that the observed differences in oral health parameters between the groups cannot be explained solely by PCOS‐related biological mechanisms, and residual confounding by unmeasured behavioral factors cannot be ruled out. The regression analyses were statistically significant but should be interpreted with caution because the sample size is modest relative to the number of predictors.

In the binary logistic regression model, the events‐per‐variable ratio of 7.2 falls below the conventionally recommended threshold of 10, raising the possibility of model overfitting and unstable coefficient estimates. The correspondingly wide confidence intervals for several variables are reflected in the reported results. The regression findings should therefore be considered exploratory and hypothesis‐generating rather than definitive and require replication in larger prospective cohorts.

### 4.8. Clinical and Public Health Implications

The findings of the present study have several implications for clinical practice and public health. PCOS affects an estimated 8%–13% of women of reproductive age globally, with prevalence estimates in India ranging from ~4% to 22.5% depending on the diagnostic criteria applied and the population studied, as summarized in the different systematic reviews [3, 4]. Given this burden and the cross‐sectional associations identified in the present study between PCOS and poorer clinical oral health and substantially reduced OHRQoL, there is a basis for considering oral health assessment and counseling as components of multidisciplinary PCOS management in future clinical guidelines, pending confirmation by longitudinal research. Similarly, whether dental professionals who identify young women with elevated DMFT, unexplained gingival inflammation, or oral health‐related psychological distress should routinely consider gynecological referral is a hypothesis that warrants prospective evaluation, particularly in populations without conventional periodontal risk factors. The most recent systematic review on this topic concluded that a collaborative approach between dental professionals and gynecologists has the potential to significantly improve women’s oral health and overall well‐being and explicitly called for the incorporation of routine periodontal screening into PCOS management [12].

The independent association of perceived psychological stress with OHRQoL within the PCOS group and the inverse association of lifestyle quality with OHRQoL in the full sample are consistent with the hypothesis that integrated psychosocial and lifestyle interventions such as stress‐management programs and structured health behavior counseling may yield oral health benefits in this population, although prospective evidence is required to confirm this. Teede et al. [2] emphasized that multidisciplinary lifestyle improvement is the primary treatment strategy in PCOS and that psychological features must be acknowledged and addressed as a prerequisite to successful management. The present study provides quantitative evidence for the extension of this principle to oral health outcomes.

## 5. Conclusion

This cross‐sectional study showed that women with PCOS had significantly poorer clinical oral health, as evidenced by higher DMFT scores and gingival bleeding and significantly lower OHRQoL across multiple domains, compared to age‐ and BMI‐matched healthy controls. The most affected domains of OHRQoL were psychological discomfort and psychological disability. This suggests that the oral health burden of PCOS extends beyond measurable tissue damage into the psychosocial domain. The associations found are cross‐sectional and should not be interpreted as evidence of causality. Exploratory regression analyses revealed that gingival bleeding and DMFT were independently associated with the PCOS status. PCOS disease status was the strongest variable associated with OHRQoL in the full sample, followed by gingival bleeding, dental caries burden, and lifestyle quality. Among the PCOS subgroup, perceived psychological stress was the only variable to be significantly and independently associated with OHRQoL, highlighting the pivotal role of psychosocial factors in determining the oral health impact in this population. Combined, these findings underscore the potential value of oral health assessment and psychosocial support in the multidisciplinary management of PCOS, pending validation in prospective longitudinal studies with larger samples, complete periodontal staging, and formal adjustment for hormonal and behavioral confounders. The present findings are in line with the vision of Sustainable Development Goal 3, ensuring good health and well‐being for all by contributing evidence in support of integrated, multidisciplinary approaches to the management of PCOS, encompassing oral health and psychosocial dimensions alongside reproductive and metabolic care.

“Future research should consider incorporating comprehensive periodontal assessments including probing depth, clinical attachment loss, and plaque index, as well as laboratory analyses of inflammatory biomarkers in gingival crevicular fluid and saliva and potentially ultrastructural tissue analyses, to more fully characterize the oral health phenotype associated with PCOS at the cellular and molecular level.”

## Author Contributions

**Jeevan V. Sambasivan**: conceptualization, methodology, investigation, data curation, formal analysis, writing – original draft. **Deepak Kumar Singhal**: conceptualization, methodology, supervision, writing – review and editing. **Nishu Singla**: conceptualization, methodology, writing – review and editing. **Jishnu Pradeep**: data curation, writing – review and editing. **Aadil Abdulhameed**: investigation, resources.

## Funding

This study did not receive any specific funding from public, commercial, or not‐for‐profit funding agencies.

## Disclosure

All authors have read and approved the final version of the manuscript. Deepak Kumar Singhal (corresponding author) had full access to all of the data in this study and takes complete responsibility for the integrity of the data and the accuracy of the data analysis. All scientific content, data, analysis, and conclusions were generated solely by the authors, and the authors take full responsibility for the integrity of the work.

## Ethics Statement

This study was approved by the Institutional Review Board, Manipal College of Dental Sciences Institutional Review Committee, and Kasturba Medical College and Kasturba Hospital Institutional Ethics Committee (IEC‐2), Manipal, India. Written informed consent was obtained from all participants prior to enrollment.

## Conflicts of Interest

The authors declare no conflicts of interest.

## Data Availability

The data that support the findings of this study are available from the corresponding author upon reasonable request. The data are not publicly available due to ethical restrictions and participant privacy obligations approved by the Kasturba Medical College and Kasturba Hospital Institutional Ethics Committee‐2, Manipal.

## References

[1] Escobar-Morreale H. F. , Polycystic Ovary Syndrome: Definition, Aetiology, Diagnosis and Treatment, Nature Reviews Endocrinology. (2018) 14, no. 5, 270–284, 10.1038/NRENDO.2018.24. 29569621

[2] Teede H. , Deeks A. , and Moran L. , Polycystic Ovary Syndrome: A Complex Condition With Psychological, Reproductive and Metabolic Manifestations That Impacts on Health Across the Lifespan, BMC Medicine. (2010) 8, 10.1186/1741-7015-8-41, 41. 20591140 PMC2909929

[3] Deswal R. , Narwal V. , Dang A. , and Pundir C. S. , The Prevalence of Polycystic Ovary Syndrome: A Brief Systematic Review, Journal of Human Reproductive Sciences. (2020) 13, no. 4, 261–271, 10.4103/JHRS.JHRS_95_18. 33627974 PMC7879843

[4] Bharali M. D. , Rajendran R. , Goswami J. , Singal K. , and Rajendran V. , Prevalence of Polycystic Ovarian Syndrome in India: A Systematic Review and Meta-Analysis, Cureus. (2022) 14, no. 12, 10.7759/CUREUS.32351, e32351. 36628015 PMC9826643

[5] Coffey S. and Mason H. , The Effect of Polycystic Ovary Syndrome on Health-Related Quality of Life, Gynecological Endocrinology. (2003) 17, no. 5, 379–386, 10.1080/09513590312331290268. 14710585

[6] Dou Y. , Xin J. , and Zhou P. , et al.Bidirectional Association Between Polycystic Ovary Syndrome and Periodontal Diseases, Frontiers in Endocrinology. (2023) 14, 10.3389/FENDO.2023.1008675, 1008675. 36755917 PMC9899846

[7] Dursun E. , Akaln F. A. , and Güncü G. N. , et al.Periodontal Disease in Polycystic Ovary Syndrome, Fertility and Sterility. (2011) 95, no. 1, 320–323, 10.1016/J.FERTNSTERT.2010.07.1052. 20800834

[8] Rathi N. and Reche A. , Risk of Periodontal Diseases in Women With Polycystic Ovary Syndrome: An Overview, Cureus. (2023) 15, no. 10, 10.7759/CUREUS.47169, e47169. 38021744 PMC10652058

[9] Varadan M. , Gopalkrishna P. , and Bhat P. V. , et al.Influence of Polycystic Ovary Syndrome on the Periodontal Health of Indian Women Visiting a Secondary Health Care Centre, Clinical Oral Investigations. (2019) 23, no. 8, 3249–3255, 10.1007/s00784-018-2741-2. 30430337

[10] Özçaka Ö. , Ceyhan B.Ö. , Akcali A. , Biçakci N. , Lappin D. F. , and Buduneli N. , Is There an Interaction Between Polycystic Ovary Syndrome and Gingival Inflammation?, Journal of Periodontology. (2012) 83, no. 12, 1529–1537, 10.1902/JOP.2012.110588. 22509751

[11] Márquez-Arrico C. F. , Silvestre-Rangil J. , Gutiérrez-Castillo L. , Martinez-Herrera M. , Silvestre F. J. , and Rocha M. , Association Between Periodontal Diseases and Polycystic Ovary Syndrome: A Systematic Review, Journal of Clinical Medicine. (2020) 9, no. 5, 10.3390/JCM9051586, 1586. 32456146 PMC7290429

[12] Angiolani F. , Gerardi D. , and Bernardi S. , et al.Bidirectional Relationship Between Periodontal Disease and Reproductive Disorders: Focus on Polycystic Ovary Syndrome, Oral. (2025) 5, no. 3, 3, 10.3390/oral5030067, 67.

[13] Bernardi S. , Marchetti E. , Torge D. , Simeone D. , Macchiarelli G. , and Bianchi S. , Ultrastructural Assessment of Human Periodontal Ligament Fibroblast Interaction With Bovine Pericardium Membranes: An In Vitro Study, Histology and Histopathology. (2025) 40, no. 8, 1185–1194, 10.14670/HH-18-860. 39723586

[14] Singla N. , Acharya S. , Prabhakar R. V. , Chakravarthy K. , Singhal D. , and Singla R. , The Impact of Lifestyles on the Periodontal Health of Adults in Udupi District: A Cross Sectional Study, Journal of Indian Society of Periodontology. (2016) 20, no. 3, 330–335, 10.4103/0972-124X.179405. 27563209 PMC4976556

[15] World Health Organization , Oral Health Surveys: Basic Methods, 2013, World Health Organization, https://www.who.int/publications/i/item/9789241548649.

[16] Slade G. D. and Spencer A. J. , Development and Evaluation of the Oral Health Impact Profile, Community Dental Health. (1994) 11, no. 1, 3–11. 8193981

[17] Acharya S. , Bhat P. V. , and Acharya S. , Factors Affecting Oral Health-Related Quality of Life Among Pregnant Women, International Journal of Dental Hygiene. (2009) 7, no. 2, 102–107, 10.1111/J.1601-5037.2008.00351.X. 19416092

[18] Cohen S. , Kamarck T. , and Mermelstein R. , A Global Measure of Perceived Stress, Journal of Health and Social Behavior. (1983) 24, no. 4, 385–396, 10.2307/2136404. 6668417

[19] Morimoto K. , Life-Style and Genetic Factors That Determine the Susceptibility to the Production of Chromosome Damage, Chromosomal Aberrations: Basic and Applied Aspects, 1990, Springer Berlin Heidelberg, 287–301.

[20] Yeniçeri Hilaloğlu N. E. and Gürsel Sürmelioğlu D. , Assessment of DMFT Indexes, Salivary Flow Rate, pH, and Detections of S.Mutans Salivary Levels by a Quantitative Real-Time PCR in Polycystic Ovary Syndrome, Cumhuriyet Dental Journal. (2022) 25, no. 2, 163–171, 10.7126/cumudj.1132273.

